# Enhanced protein adsorption upon bulk phase separation

**DOI:** 10.1038/s41598-020-66562-0

**Published:** 2020-06-25

**Authors:** Madeleine R. Fries, Daniel Stopper, Maximilian W. A. Skoda, Matthias Blum, Christoph Kertzscher, Alexander Hinderhofer, Fajun Zhang, Robert M. J. Jacobs, Roland Roth, Frank Schreiber

**Affiliations:** 1 Institute for Applied Physics, Auf der Morgenstelle 10, University of Tübingen, 72076 Tübingen, Germany; 2 Institute for Theoretical Physics, Auf der Morgenstelle 14, University of Tübingen, 72076 Tübingen, Germany; 3 ISIS Neutron and Muon Source, Science and Technology Facilities Council, Rutherford-Appleton Laboratory, Didcot, OX11 0QX United Kingdom; 4 Surface Analysis Facility, Chemistry Research Laboratory, Department of Chemistry, University of Oxford, 12 Mansfield Road, Oxford, OX1 3TA United Kingdom

**Keywords:** Biomaterials, Soft materials, Biological physics

## Abstract

In all areas related to protein adsorption, from medicine to biotechnology to heterogeneous nucleation, the question about its dominant forces and control arises. In this study, we used ellipsometry and quartz-crystal microbalance with dissipation (QCM-D), as well as density-functional theory (DFT) to obtain insight into the mechanism behind a wetting transition of a protein solution. We established that using multivalent ions in a net negatively charged globular protein solution (BSA) can either cause simple adsorption on a negatively charged interface, or a (diverging) wetting layer when approaching liquid-liquid phase separation (LLPS) by changing protein concentration (*c*_*p*_) or temperature (*T*). We observed that the water to protein ratio in the wetting layer is substantially larger compared to simple adsorption. In the corresponding theoretical model, we treated the proteins as limited-valence (patchy) particles and identified a wetting transition for this complex system. This wetting is driven by a bulk instability introduced by metastable LLPS exposed to an ion-activated attractive substrate.

## Introduction

Controlling and understanding protein adsorption is key to a number of phenomena in biomaterial science and medical devices such as biocompatibility, osseointegration, inflammation and contamination^1–3^. One way to systematically study the underlying interaction mechanisms between proteins and solid surfaces is to alter the surface chemistry and topography e.g. through the use of alloys of different composition, self-assembled monolayers (SAMs), membrane bilayers, polymer brushes, smart biomaterials or tissue engineering^1,4–7^. An interesting, and in fact efficient, alternative to modifying the surface properties would be to tune protein adsorption by exploiting suitable thermodynamic conditions, i.e. conditions that favour a certain level of adsorption driven by the underlying bulk phase behaviour.

Adsorption at solid-liquid interfaces is the result of sufficiently attractive substrate-fluid and intermolecular fluid interactions. Strongly enhanced or macroscopic adsorption may in particular result in the vicinity of bulk instability regions, a phenomenon called ‘wetting’ that is mostly explored in the statistical physics of ‘simple liquids’^8–10^. Although the bulk phase behaviour of protein solutions shares intriguing similarities with that of suspensions of spherical colloids^11–14^, it is not clear a priori to what extent surface phenomena such as wetting can be transferred to solutions of proteins, in view of their significant complexity and patchy nature^15–22^. Furthermore, the tailoring of adsorption *beyond* the monolayer would be of significant importance for the understanding of e.g. heterogeneous nucleation of crystals or for improving the biocompatibility of implants by pre adsorption, which makes this study not only important fundamentally, but also for applications.

**Figure 1:**
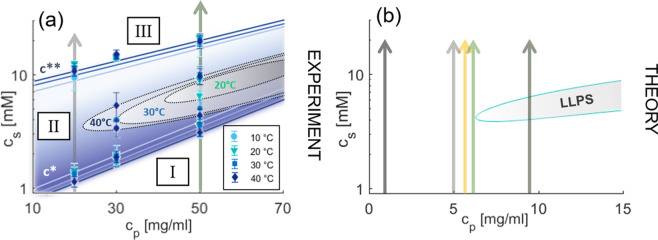
Phase diagram. (**a**) Experimental phase diagram of BSA (*c*_*p*_) and YCl_3_ (*c*_*s*_) for various temperatures *T*. The lines c^*^ and c^**^ determine the boundaries of the region where the solution is turbid and dominated by large protein clusters (blue area, regime II). It broadens slightly with increasing *T*. The vertical arrows indicate the paths taken in the adsorption experiments. The LLPS region (gray-shaded areas) starts to occur at 20 °C and quickly broadens with increasing temperatures. Note that the gray-shaded areas do not display the coexisting densities but the regions at which LLPS was observed. The experimental measured values of the phase boundaries can be found in Table S1. (**b**) Theoretical phase diagram of BSA (*c*_*p*_) and YCl_3_ (*c*_*s*_) based on theoretical DFT calculations in ref. ^20^. The obtained phase diagram does not contain an explicit temperature dependency, but can be compared to the experimental 20 °C data. The vertical arrows indicate the paths taken in the calculations for Fig. 3. Note that due to the intentionally simplified nature of the model with only few parameters the agreement with experiment is only semi-quantitative.

Salts provide a versatile way to manipulate the interactions. Specifically, *multivalent* ions can induce novel effects at interfaces, going well beyond mean-field behaviour^23^, such as strong coupling through ion-ion correlations^24,25^, micelle formation at liquid-liquid interface transitions^26^, elemental selectivity at interfaces^27^ and charge inversion in phospholipids^28^, in polyelectrolytes^29^, and DNA complexes^25^. In polyelectrolyte brushes, multivalent ion bridging was found to cause diminishing lubrication properties^30^ illustrating the versatile role multivalent ions can have. For proteins, multivalent ions have also proven to be a powerful tool in inducing a broad variety of interactions and associated phase behaviour^31,32^. In order to provide a basis for the adsorption studies, we will first explain the *bulk* behaviour of protein solutions. The phase diagram of bovine serum albumin (BSA) and yttrium chloride (YCl_3_) in water is shown in Fig. 1(a). BSA in aqueous solution is net negatively charged at neutral pH^33^. YCl_3_ is particulary suitable as a model salt since it has relatively weak effects on the pH compared to other multivalent salts. The addition of multivalent salts, such as YCl_3_, to the BSA solution screens repulsive electrostatic forces due to binding of multivalent cations to the protein surfaces. At the same time, this ion-binding process induces highly directional attractive protein-protein interactions, resulting in the formation of protein bridges mediated by cations^20^. At sufficiently low salt concentrations *c*_*s*_, the solution remains clear (regime I). If *c*_*s*_ is increased, protein clusters give rise to a transition from a clear to a turbid solution (regime II) after crossing a boundary denoted by c^*^. Importantly, regime II also features a (metastable) closed-loop liquid-liquid phase separation (LLPS) into a protein-poor and protein-rich phase, which we will exploit in this study and which can also facilitate protein crystallization^32,34,35^. Upon further increasing *c*_*s*_, the system undergoes charge inversion, causing the protein clusters to dissolve. This process is known as re-entrant condensation (RC), which is defined by a second transition (c^**^) from a turbid to a clear solution (regime III)^31,36^. We note that the concept of charge inversion is not unique to BSA, but was shown for several proteins, e.g. BLG^31,35,37^. The LLPS phase boundaries depend on the solution temperature *T*, as indicated in Fig. 1(a).

Several aspects of the phase behaviour of proteins in solution can be understood with the statistical physics of colloidal fluids^11,12^. In particular, models that treat proteins as patchy particles, interacting *via* highly-directional forces, turned out to be very successful^15–19,21,38,39^. Anisotropy in protein-protein interactions is the result of, *inter alia*, non-uniformly distributed surface charges, presence of hydrophobic and hydrophilic zones on the protein surface, or the formation of hydrogen bonds. The behaviour of proteins in the presence of multivalent salt can be successfully modelled as patchy colloids, where the patches are activated by cations^20^: In addition to a hard-sphere-like core repulsion, a patch-patch interaction is mediated by ions which can activate the sites by chemically binding to the protein surface; a bond between two distinct proteins is then only possible if an activated patch meets a deactivated one (see Methods for further details). The resulting phase diagrams, which can be obtained from Wertheim’s perturbation theory for associating particles^40–42^, are in excellent qualitative agreement with the experiments considering the coarse-grained nature of the model. This includes RC in terms of protein clusters and a closed-loop LLPS region. In line with experiments^32^, critical protein volume fractions are predicted to occur at values below 10%, and volume fractions of the high-density protein phase around 20%. This is indeed a prominent feature of patchy fluids^43^, and cannot be understood with fluids interacting *via* isotropic forces where liquid densities often reach volume fractions of 40% or beyond^10^.

In this paper, we demonstrate the control of enhanced adsorption *beyond* a monolayer and explore if the wetting phenomena, known from simple fluids, can also be exploited in the rather complex system of a protein solution exhibiting LLPS in the presence of multivalent salts in order to induce enhanced protein adsorption at a planar interface. The wetting is driven by a combination of underlying bulk thermodynamics and sufficiently strong salt-induced wall-protein attractions. We discuss the experimental and theoretical implications and potential applications in a broader context of wetting by patchy particles, for which the present system is an interesting realization.

## Results

We study the adsorption of an aqueous BSA solution in the presence of YCl_3_ at a silicon dioxide (SiO_2_) interface^44^ as a function of *c*_*s*_ experimentally by means of ellipsometry and quartz-crystal microbalance with dissipation (QCM-D) (for details on Methods see^45^) and theoretically within the framework of classical density functional theory (DFT). By comparing the experimental data with the theoretical predictions in a *simplified* model system, we obtain a clearer insight into the behaviour of the *complex* protein system.

The general bulk behaviour of globular proteins and multivalent ions has been well studied over the past decade^31,32,34,36,46,47^. As a first step, we have determined the bulk phase behaviour of this specific system and its temperature dependence. This is an essential key in order to draw meaningful conclusions regarding the connection between adsorption and bulk thermodynamics. The experimental results are summarized in Fig. 1(a) while the theoretical phase diagram calculated within the model of ion-activated patchy particles^20^ is shown in Fig. 1(b). For 10 °C, which is below the lower solution critical temperature (LCST), no coexistence of protein-poor and protein-rich phase is found. At higher temperatures (20 °C, 30 °C, and 40 °C), the LLPS region (gray areas) broadens strongly. Interestingly, the lines c^*^ and c^**^, defining regime II, do not show such a strong temperature dependency. While there are also pH-related effects (see Fig. S4 of the Supporting Information), pH is not driving the rich phase behaviour for the salts employed here^33^. Absolute numbers of the measured phase boundaries can be found in Table S1^45^.

In the adsorption experiment, we can approach the LLPS region from low protein concentrations *c*_*p*_ by either increasing *c*_*p*_, or increasing *T*. The latter procedure is achievable as the LLPS region for the present system is bounded by LCST and broadens with increasing *T*^48^.

### Adsorption upon approaching LLPS by varying ***c***_***p***_

**Figure 2:**
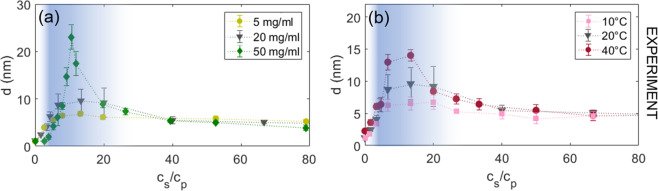
Ellipsometric protein adsorption measurements. (**a**) Protein layer thickness *d* versus *c*_*s*_/*c*_*p*_ upon approaching the LLPS by changing the protein concentration (*c*_*p*_ = 5 mg/ml, 20 mg/ml and 50 mg/ml) obtained from ellipsometry measurements at 20 °C. (**b**) Protein adsorption as function of *c*_*s*_/*c*_*p*_ upon approaching the LLPS by changing *T* (10 °C, 20 °C and 40 °C) *via* ellipsometry at 20 mg/ml. The adsorption curves show an increase in *d* by increasing *c*_*p*_ or *T* in regime II (blue-shaded). Regimes I and III are essentially unaffected by the change in *c*_*p*_ or *T*.

The most intuitive way to approach the LLPS region experimentally is to change *c*_*p*_. We studied the adsorption behaviour at three different values of *c*_*p*_, namely 5 mg/ml, 20 mg/ml and 50 mg/ml at fixed *T* = 20 °C and varied the value of *c*_*s*_ as indicated by the arrows in Fig. 1(a) (the path for *c*_*p*_ = 5 mg/ml is far below the LLPS and not shown). The resulting effective adsorbed protein layer thickness *d* in nm (for definition see Methods section) are displayed in Fig. 2(a) as a function of *c*_*s*_/*c*_*p*_. The turbid regime II is indicated by the shaded area. The normalized abscissas allows us to show the adsorption profiles for different values of *c*_*p*_ in one figure, although c^*^ and c^**^ increase with increasing *c*_*p*_ (see Fig. 1(a)).

SiO_2_ in contact with water is negatively charged. Therefore, without added salt, only few proteins are adsorbed at the interface due to dominating repulsive electrostatic forces between the negatively charged surface, and the net negatively charged BSA molecules at neutral pH^49–51^. This results in adsorption limited to sub-monolayers^52^. Ellipsometry measurements show a fitted effective thickness of *d* ≈ 1 nm (BSA has an effective sphere radius of *R*_*p*_ ≈ 3.5 nm, i.e. the coverage is below 1 full monolayer (ML)).

When increasing *c*_*s*_, the repulsive electrostatic forces are screened, and attractive interactions dominate in regime II. The ions adsorbed at the interface exert a strongly attractive force on the proteins^44^. The attractive protein-protein interaction in addition to the attractive substrate cause *d* to exhibit a prominent maximum at *c*_*s*_/*c*_*p*_ ≈ 10. For higher *c*_*s*_ entering regime III, *c*_*s*_ > c^**^, the adsorption curves feature a re-entrant effect for all *c*_*p*_. Here, the dominating protein-protein interactions become again more repulsive^31^, while the substrate remain attractive. As a consequence, *d* decreases down to a plateau with a value of ~5 nm corresponding to roughly 1 ML of proteins that remain adsorbed at the wall (1 ML: *d* = 4 nm). Remarkably, this appears to be insensitive to *c*_*p*_.

Importantly, the adsorption maximum *d*_max_ increases strongly from *d* = 6.8 ± 0.51 nm at *c*_*p*_ = 5 mg/ml, to 9.6 ± 2.5 nm at 20 mg/ml, and to 23.5 ± 2.55 nm at *c*_*p*_ = 50 mg/ml. Note that for *c*_*p*_ = 50 mg/ml the system is already phase-separated, and only the dilute (protein-poor) phase was used for the adsorption measurement, to avoid interfacial effects between the dilute and dense protein phase, or density gradients influencing the measurement. The non-linear increase of *d*_max_ indicates that this is not simply the result of more proteins being present in the system, but rather is related to the bulk phase behaviour. We therefore suggest that the strong adsorption peak at *c*_*p*_ = 50 mg/ml may be the precursor of a ‘wetting’ transition.

**Figure 3:**
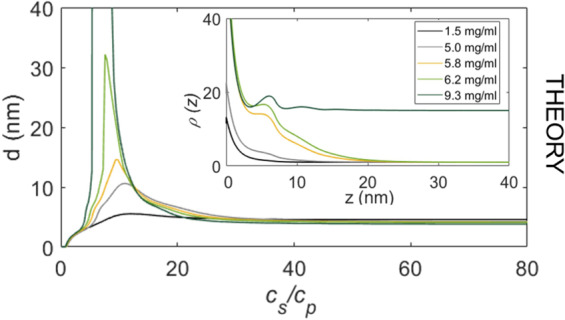
DFT calculations. Protein layer thickness *d* versus *c*_*s*_/*c*_*p*_ as obtained within DFT for different paths through the theoretically calculated phase diagram (Fig. 1(b)). The theoretical predictions of *d* agree qualitatively very well with those from experiments shown in Fig. 2(a), except for the path crossing the LLPS region. The divergence in the theory is due to the grand canonical ensemble. Note that due to the complexity of the system the canonical ensemble does not quantitatively describe the experiment. (*inset*) Protein density *ρ*(*z*) normalized to its value for *z* → ∞ corresponding to the adsorption maxima. When the LLPS region is crossed on the protein poor side, a macroscopically thick film of the protein rich phase can be found adsorbed at the attractive substrate (dark green line, *c*_*p*_ = 9.3 mg/ml).

Using our model of activated patchy particles, the re-entrant adsorption trend can be explained within the framework of DFT^53,54^, where the re-entrant behaviour was shown to be a direct consequence of the underlying bulk interactions of the proteins^44^. Details of the theoretical modelling are given in the Methods section. We now employ this model to investigate the behaviour of protein adsorption upon approaching the LLPS region. The theoretical predictions for *d*, as a function of *c*_*s*_/*c*_*p*_, are displayed in Fig. 3 for several fixed *c*_*p*_ (corresponding to the arrows in Fig. 1(b)).

In excellent agreement with our experimental results, the model predicts that the adsorption in regime II is enhanced strongly upon approaching the LLPS region. For the path crossing the LLPS (dark green arrow), the attractive substrate becomes covered by a macroscopic film of the coexisting high-density protein phases, i.e. *d* diverges. The corresponding protein density profiles *ρ*(*z*) at the maxima of *d* obtained from DFT are shown in the inset of Fig. 3 evidence the divergence in the dark green curve (*ρ* = *ρ*_*liquid*_ for *z* → ∞). Note that the curves are normalized with respect to the bulk value of the protein-poor phase. We find that for the path that misses the LLPS region slightly, the maximum of *d* corresponds to a film with the density of the protein-rich phase that is a few protein diameters thick. When the chosen path crosses the LLPS region at the protein-poor phase, wetting theory of simple liquids^8^ would predict that if the substrate is sufficiently attractive, *d* can diverge, i.e. a macroscopically thick film of the coexisting protein-rich phase can be adsorbed at the substrate. Our DFT model for proteins as ion-activated patchy particles predicts a wetting behaviour which, interestingly, does not differ significantly from results for wetting of fluids with isotropic potentials despite the much higher complexity and the fundamentally different interactions.

### Adsorption upon approaching LLPS by varying ***T***

Our system offers an interesting, independent way to experimentally demonstrate the increase of *d* and relate this to the onset of a wetting transition: We can enter the LLPS regime at constant *c*_*p*_ by changing *T*^48^. Due to the LCST behaviour, increasing *T* up to 40 °C leads to a significant expansion of the region where LLPS is found (cf. Fig. 1(a)). Ellipsometric measurements of the adsorption layer were performed at 10 °C, 20 °C and 40 °C at *c*_*p*_ = 20 mg/ml.

The resulting adsorption profiles shown in Fig. 2(b) exhibit a similar behaviour as those in Fig. 2(a): By increasing *T*, *d* clearly increases in regime II. We note that the increase is not as strong as for the previous path varying *c*_*p*_, which most likely is related to the fact that for *T* = 40 °C the path at *c*_*p*_ = 20 mg/ml is still located well away from the LLPS boundary. Nevertheless, these results strongly suggest that the observed protein adsorption is connected to the underlying bulk phase diagram. This is further supported by considering the adsorption behaviour in regime I and III relative to that in regime II. Here, the values of *d* are virtually unaffected when changing *T*, even on an absolute scale. In regime I (up to *c*_*s*_/*c*_*p*_ ≈ 5), the data is nearly indistinguishable, and in regime III, all curves simultaneously converge to a plateau of *d* ≈ 5 nm (as in the previous measurements where *c*_*p*_ was varied). Thus, the adsorption behaviour in regime I and III seems to be mainly guided by surface properties and is independent of (temperature-dependent) bulk conditions. In contrast, in regime II, adsorption is enhanced due to the combination of the ion-activated-attractive interface and the bulk instability.

### Complementary measurements and properties of adsorbed layer

**Figure 4:**
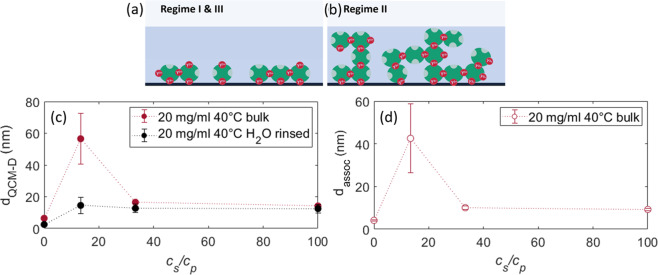
Properties of adsorbed layer. (*Top*) Sketch illustrating the different layer formation of (**a**) a (monolayer) adsorption layer compared to (**b**) a thicker (wetting) layer. (**c**) QCM-D measurements of the adsorbed amount of proteins at the solid-liquid interface at *c*_*p*_ = 20 mg/ml and 40 °C. It illustrates the adsorption behaviour and its dependence on *c*_*s*_. The black data points (*d*_*rinsed*_) show *d*_*QCM*−*D*_ after flushing the cell with H_2_O, thus, the irreversibly bound proteins. (**d**) Associated water (*d*_*assoc*_). Since the QCM-D detects the adsorbed proteins plus its associated water, whereas ellipsometry fits the data to a volume fraction of 1, which is laterally averaged over the measured surface. Through the subtraction of *d*_*EM*_ from *d*_*QCM*−*D*_, the associated water surrounding the proteins can be calculated (usually this is rather illustrated with the difference in mass than thickness).

We extended the adsorption study with complementary QCM-D. The results are shown in Figs. S5 and 4(c) for *T* = 20 °C and 40 °C, respectively. Importantly, the QCM-D data exhibits the same trend as the previous results: The adsorption maximum in regime II increases with increasing temperature and the curves approach a plateau value in regime III. The difference in absolute values of *d*_*QCM*−*D*_ obtained from QCM-D relative to that of ellipsometry arises since the QCM-D measures in addition to the protein in a relatively open (sponge-like) morphology, also the water directly associated with these adsorbed proteins, whereas ellipsometry measures an effective *d* by assuming a step function for the protein density profile with a volume fraction of 1, which is laterally averaged over the measured surface. This associated water includes the hydration layer, hydro-dynamically bound water and trapped water in a presumably sponge-like morphology of the adsorbed protein layer. Through the subtraction of *d*_*EM*_ from *d*_*QCM*−*D*_, the amount of associated water *d*_assoc_ within *d*_*QCM*−*D*_ surrounding the proteins can be calculated in Fig. 4(d).

This provides valuable insight into the density and the structure of the formed layer. It turns out that in regime II the amount of water contained in the layers is about two times higher at *T* = 20 °C (Fig. S5), and four times higher at 40 °C, respectively, compared to regimes I and III indicating the formation of a diffuse layer in the dense-liquid regime II. This information allows us to extend the picture of enhanced adsorption. In regime II, there are indeed more proteins adsorbed, thus “enhanced adsorption”, but at the same time this layer has significantly more water associated to it. This means that this enhanced protein layer is more diffuse than a “normal” (densely packed) adsorption layer due to the water uptake, while also having more proteins incorporated leading to a different layer morphology. The different configuration of the adsorption layer vs. wetting layer is illustrated in Fig. 4(a,b). Interestingly, this is consistent with the trend of the dissipation parameter *D* (see Fig. S3 and S4) obtained from the QCM-D measurements. *D* is a measure for the viscoelastic properties of the adsorbed layer, where a higher value of *D* means that the layer is ‘softer’ and more diffuse^55^ and a small value of *D* assumes a solid-like, rather stiff adsorbed layer.

We also studied the reversibility of the adsorption process from the QCM-D results. When the system is rinsed with water after the adsorption measurements, we find that roughly 1 ML (≈12 nm *including* its coupled water for QCM-D) remains adsorbed at the substrate (black solid data points in Fig. 4). In addition, a decrease of the value of *D* is observed, corresponding to a ‘stiff’ layer^55^. Furthermore, for *c*_*s*_ corresponding to regime II, no increase in adsorption is observed any more. This observation indicates that a layer of proteins is irreversibly bound at the substrate with stronger interaction than the excess proteins above with the substrate.

## Discussion

Our experimental and theoretical results suggest that the enhanced protein adsorption upon approaching LLPS features the onset of a ‘wetting’ transition (i.e. the formation of a macroscopically thick protein layer) caused by dominatingly attractive protein-protein and protein-substrate interactions, both of which are mediated by the multivalent ions. In experiments with independent complementary methods (ellipsometry and QCM-D), we have demonstrated that this effect can be achieved *via* two independent pathways, namely approaching the LLPS region by varying *c*_*p*_ and by varying *T*.

The observation of strongly enhanced adsorption is particularly striking, since it underlines that interfacial phenomena such as wetting, which are known from the statistical physics of simple liquids, may also be found in rather complex solutions of proteins. This provides promising new perspectives for controlling protein adsorption at interfaces. In fact, exploiting the underlying bulk phase behaviour and thermodynamic conditions offers a particularly efficient tool for tailoring a desired protein density at substrates in a controlled manner (not limited to BSA), which is relevant for many biological or medical applications such as biosensors or better biocompatibility in dental implants, lenses and joints and might be extended to other biological systems such as DNA nano-stars used in hydro-gels^56^. The consequences are important for the control and tailoring of protein adsorption and possibly at some stage (heterogeneous) nucleation of protein crystals or other high-density phases at the solid-liquid interface. We also emphasize that in particular the use of multivalent ions represents a versatile tool for experimentally tuning substrate-protein interactions to achieve a desired level of protein adsorption. Moreover, adsorption phenomena only rarely seem to have been investigated from the perspective of patchy colloids^57^ for which proteins are a promising experimental realization.

## Methods

The study was designed as a combination of adsorption experiments with a number of complementary tools (see below) and independent theoretical calculations (DFT; see below). Details of the calculations are provided in this section, as are the experimental details. Each experimental data point was independently confirmed both by repeating the measurement as well as cross-checking with an independent technique (comments on error bars see below). The remarkably good agreement between experiment and theory with an intentionally small number of phenomenological parameters for this rather complex system was considered as evidence of having established the generic and general nature of the effect of adsorption enhancement upon bulk phase separation.

### Materials and bulk phase behaviour

The materials used in this study were purchased from Sigma Aldrich, namely BSA with a purity of ≥98% (product No. A7906) and YCl_3_ with a purity of 99.99 % (product No. 451363). SA is one of the most abundant blood proteins in mammals and has a net negative charge of −10e at neutral pH^46^. The protein solutions were prepared with degassed Milli-Q water at *c*_*p*_ of 5, 20, and 50 mg/ml and *T* ranging from 10 to 40 °C. *c*_*s*_ was varied from 0 to 60 mM. While in this study we focus on BSA, we emphasize that the effects are expected to be rather general, as indicated in tests with, e.g. BLG.

The temperature-dependent phase diagram shown in Fig. 1(a) was generated with the Thermostat C of Eppendorf for a stable temperature control. The phase transitions were determined by eye based on onset of turbidity in Fig. S2. Typical aggregation sizes and its formation was part of a previous study by Soraruf *et al*.^47^. For the lowest investigated protein concentration of *c*_*p*_ = 5 mg/ml, c^*^ and c^**^ had to be determined *via* UV-Vis spectroscopy measurements with the Cary 50 UV-visible spectrometer of Varian Technologies since the turbidity detection by eye was not sufficiently precise, see Fig. S2^45^. The spectrometer was also used to perform absorbance measurement to determine the protein stock solution concentration *via* the Lambert-Beer law.

### Interface studies/adsorption

All characterization was performed *in situ* and the adsorption time was set to 1 h, which was sufficiently close to equilibrium conditions. Ellipsometric measurements were performed with a Woollam VASE M-2000 and a Beaglehole Picometer at the solid-liquid interface with a home-made cell at the Brewster angle of SiO_2_ at 68°. Standard Si wafers with a native oxide layer and a (111) orientation were utilized as substrates. For data analysis, the CompleteEASE software of J. A. Woollam was used to create a model taking into account the optical constants of the individual layers. The BSA adsorption layer was fitted with a Cauchy layer of A = 1.43, B = 0.01. To quantify the adsorption, we used the effective measured *d* assuming a volume fraction of 1, which is laterally averaged over the measured surface.

Complementary studies were conducted with the quartz-crystal microbalance with dissipation (QCM-D), Q-Sense Analyzer, of Biolin Scientific^51,58^. This set-up allowed for measurements with the substrate on top of the solution excluding sedimentation effects. SiO_2_-coated quartz sensors (product No. QS-QSX303) were used for the adsorption measurements.

More details on the methods, experimental conditions and original data can be found in the *Supplementary Information* in Table S2 and Figs. S3–S5^45^.

All measurements were repeated at least three times to ensure their reproducibility and to estimate real standard deviations as statistical error bars. The systematic errors (e.g. wavelength and angle calibration of the ellipsometer) are substantially smaller. In all figures, the mean value of those measurements with real standard deviation is plotted.

### Theoretical model

Based on the Wertheim perturbation theory for fluids of patchy particles^40–42,59^, the present model considers proteins as hard spheres with radius *R*_*p*_ and *M* distinct and independent binding sites (patches). These sites can be occupied by salt ions (Y^3+^), thereby activating a given patch (ion binding). The occupation probability of a site reads^20^

1 $$\Theta ({\mu }_{s})={(1+\exp (\beta {\varepsilon }_{b}-\beta {\mu }_{s}))}^{-1},$$

where *μ*_*s*_ denotes the salt chemical potential, *β* = (*k*_*B*_*T*)^−1^ the inverse temperature, and *ε*_*b*_ the binding energy between the site and the ion. A bond between two patches of distinct proteins is possible only if an activated patch meets a de-activated one (and thus forms an ion bridge). The energy of a protein-protein bond is given by^20^

2 $$\beta {\varepsilon }_{pp}({\mu }_{s})=\beta {\varepsilon }_{uo}\Theta ({\mu }_{s})(1-\Theta ({\mu }_{s})),$$

in which *ε*_*uo*_ defines an energy scale for the interaction between the occupied and unoccupied site. The longer-ranged electrostatic repulsion which dominates in the system without salt (regime I) and very high salt concentrations (regime III) is effectively described *via* the hard-sphere repulsion between proteins. Note that the model accounts for the salt ions implicitly *via* the chemical potential *μ*_*s*_, which implies that the total salt concentration *c*_*s*_ as a function of *μ*_*s*_ cannot be predicted self-consistently within this approach. We therefore make use of the location of the minimum of the experimentally determined second virial coefficient ${B}_{2}/{B}_{2}^{HS}$ at *c*_*p*_ = 20 mg/ml in order to calibrate *c*_*s*_(*μ*_*s*_)^44^.

We employ classical density functional theory (DFT)^53^ providing a powerful and well-established framework to investigate inhomogeneous density distributions in any external potential. The key statement of DFT is the theorem that a functional

3 $$\Omega [\rho ]= {\mathcal F} [\rho ]+\int {\rm{d}}{\bf{r}}\,\rho ({\bf{r}})[{V}_{{\rm{ext}}}({\bf{r}})-\mu ]$$

of the inhomogeneous density profile *ρ*(*r*) exists and takes its minimum, the grand potential, at the equilibrium density distribution^53^. We employed a DFT formulation of the Wertheim theory^60^ based on fundamental measure theory (FMT) for hard spheres^54,61^, to calculate *d* at the SiO_2_-water interface. Effectively, this we describe by a salt-dependent short-ranged wall-protein potential *V*_wp_(*z*) where *z* is the distance normal to the substrate. The model predicts a non-monotonic, re-entrant adsorption layer at the substrate depending on *c*_*s*_ in excellent accordance with experiments^44,62^. For more details on the underlying bulk model and the ion-activated attractive protein adsorption model, we refer refs. ^20,44^.

## Supplementary information

Supplementary Information.

## Data Availability

The experimental data is available from the corresponding author on reasonable request.

## Code availability

The code used for the DFT calculation is available from the corresponding authors upon reasonable request.
