# Supplementary material for: Enhanced protein adsorption upon bulk phase separation
Source: Sci Rep. 2020 Jun 25;10:10349. doi: 10.1038/s41598-020-66562-0 (PMC7316800; doi:10.1038/s41598-020-66562-0)
Supplement: Supplementary file 1 — Supplementary Information. [file 41598_2020_66562_MOESM1_ESM.pdf]

[a] These authors contributed equally to this work: M.R.F. and D.S. * Correspondence and requests for materials should be addressed to R.R. (email: roland.roth@uni-tuebingen.de) or to F.S. (email: frank.schreiber@uni-tuebingen.de)

Supplementary Information : Enhanced protein adsorption upon bulk phase separation

Madeleine R. Fries[1,a], Daniel Stopper[2,a], Maximilian W. A. Skoda[3], Matthias Blum[1], Christoph Kertzscher[1], Alexander Hinderhofer[1], Fajun Zhang[1], Robert M. J. Jacobs[4], Roland Roth[2,*] and Frank Schreiber[1,*]

[1] *Institute for Applied Physics, Auf der Morgenstelle 10, University of Tübingen, 72076 Tübingen, Germany*

[2] *Institute for Theoretical Physics, Auf der Morgenstelle 14, University of Tübingen, 72076 Tübingen, Germany*

[3] *ISIS Neutron and Muon Source, Science and Technology Facilities Council, Rutherford-Appleton Laboratory, Didcot, OX11 0QX, United Kingdom and*

[4] *Surface Analysis Facility, Chemistry Research Laboratory, Department of Chemistry, University of Oxford, 12 Mansfield Road, Oxford, OX1 3TA, United Kingdom*

(Dated: May 5, 2020)

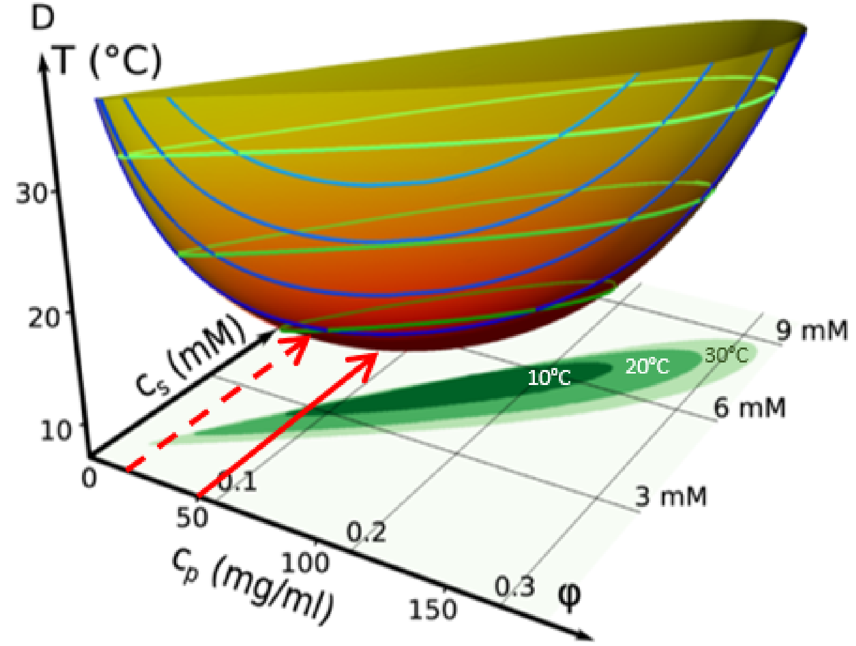

Figure 1: **Temperature-dependent phase behaviour.** 3D schematic of the LCST-LLPS regime of the BSA/YCl_3 system (modified from Ref. [1]).

Here, the experimental details to the methods used and additional data to our experiments studying the divergent thickness effect with *multivalent ions* are shown, supporting our main finding of a wetting transition.

DETERMINATION OF THE TEMPERATURE-DEPENDENT PHASE BEHAVIOUR OF BSA/YCL_3

The phase behaviour of BSA/YCl_3 is characterized by two phase transitions c^* and c^{**} and a metastable liquid-liquid phase separation (LLPS) regime induced by a lower critical solution temperature (LCST, Fig. 1).

Regime II (the regime in between the two phase transitions) is defined by cluster formation, thus its physical appearance changes from a clear to turbid solution. This transition from clear to turbid and turbid to clear can be detected by eye in Fig. 2, if the protein concentration c_p is high enough. Hereby, c^* has an abrupt change, whereas c^{**} is smeared out and thus is determined through taking the median of the last turbid to the first clear solution. LLPS is also detectable by eye since over time a dense yellowish liquid is separating

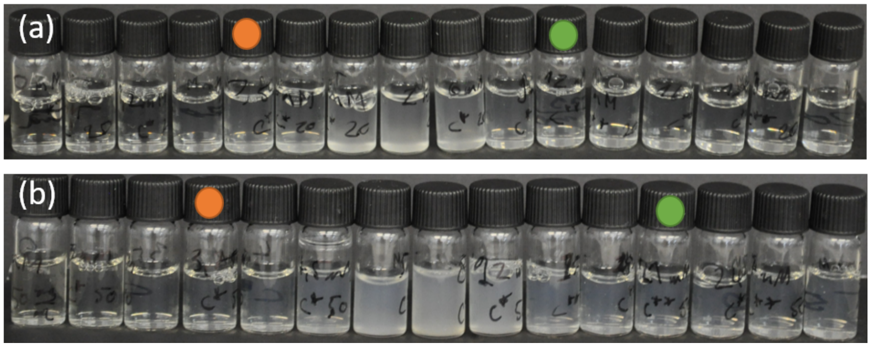

Figure 2: **Dilution series.** Images of BSA/YCl_3 solutions at 20°C and at different c_p of (a) 20 mg/ml and (b) 50 mg/ml BSA at varying c_s of 0-30 mM. The orange circles mark c^* and the green ones c^{**}, respectively.

Table I: **Phase behaviour of BSA and YCl_3.** Phase transition c^*, c^{**}, and LLPS in mM at different protein concentrations and temperatures.

**Temperature**	**10°C**		**20°C**				**30°C**				**40°C**			
c_p [mg/ml]	c^*	c^{**}	c^*	c^{**}	lower LLPS	upper LLPS	c^*	c^{**}	lower LLPS	upper LLPS	c^*	c^{**}	lower LLPS	upper LLPS
20	1.33	9.33	1.37	12.5	-	-	1.33	11.17	-	-	1.13	10.67	-	-
30	2	14	1.8	14.17	-	-	1.73	14.17	3.33	4	1.87	15.17	3.33	5.33
50	3.67	18.5	3.53	21	6.5	9	3.53	20.17	5	10	3.07	19.5	4.33	9.67

from the dilute phase (clear) [2]. The absolute numbers to those transitions can be found in Tab. I and Fig. 1(a) (main text).

In the case of 5 mg/ml c_p, c^* and c^{**} have to be determined *via* UV-Vis spectroscopy transmission measurements with the Cary 50 UV-visible spectrometer of Varian Technologies since the turbidity detection by eye was not sufficiently precise. Here, c^* is defined by the drop in transmission and c^{**} is determined by taking the median of the minimum in transmission to the plateau value, see Fig. 3.

*p*H MEASUREMENTS OF BULK SOLUTION

In globular proteins and in general in the context of charged particles, pH is an important parameter. In our case, though, using YCl_3, the overall behaviour is driven by multivalent

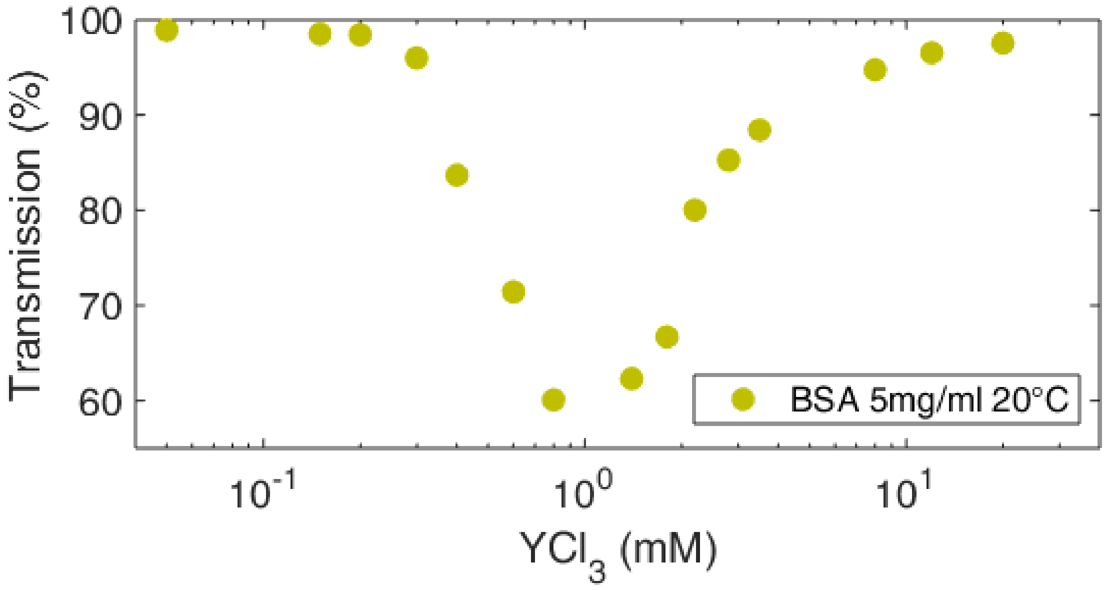

Figure 3: **Phase transition at low salt concentrations.** UV-Vis transmission measurement at 5 mg/ml c_p at 20°C. The phase transition c^* and c^{**} are defined by the decrease and increase in transmission.

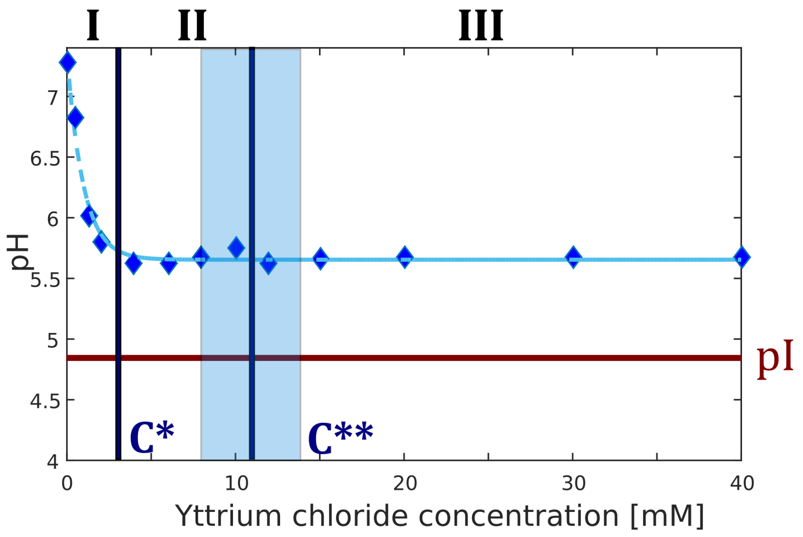

Figure 4: **Bulk properties.** pH-meter measurements of the bulk protein solution of 20 mg/ml BSA at 20°C by varying YCl_3 concentration.

ions, and only small pH changes occur. As an example, pH measurements at 20 mg/ml c_p are shown in Fig.4. This is consistent with previous studies of our group [3].

ADDITIONAL INFORMATION ON SAMPLE PREPARATION

Solution preparation

The BSA/YCl_3 solution were prepared 30 min before protein adsorption measurements were conducted to allow the solution to stabilise.

Substrate preparation

For ellipsometry, all substrates were cleaned with acetone, isopropanol and water each for 5 min in the ultrasonicator. For QCM-D, all substrates were cleaned ex situ with acetone, isopropanol and water each for 5 min in the ultrasonicator and 10 min ozone cleaned. In situ, the substrates were cleaned with 2% Hellmanex, isopropanol and water.

ADDITIONAL INFORMATION ON QUARTZ-CRYSTAL MICROBALANCE WITH DISSIPATION (QCM-D)

Complementary studies were conducted with the quartz-crystal microbalance with dissipation (QCM-D), Q-Sense Analyzer, of Biolin Scientific. This set-up allowed for measurements with the substrate on top of the solution excluding sedimentation effects. SiO_2-coated quartz sensors (product No. QS-QSX303) were used for the adsorption measurements. The microbalance was calibrated in water prior to the adsorption measurement. Afterwards, the cell was exchanged with the prepared protein/salt solution and the adsorption process was measured for one hour. Then, the cell was flushed with water to check for the reversibility of the adsorption process.

The raw measured data consists of two parameters: frequency F and dissipation D. An example for it is plotted in Fig. 5. Already from those two parameters, one can get information about the properties of the adsorbed layer. The higher F, the more proteins/mass is adsorbed on the interface. The higher D, the more diffuse and viscoelastic the adsorbed layer. D values below $2e^{-6}$Hz are assumed to belong to a rather stiff layer and above to a more diffuse layer [4]. During adsorption, a more diffuse layer is formed in regime II than in the other regimes. Thus, the thicker the adsorbed layer, the more diffuse it is. After rinsing with H_2O, all adsorbed films loose all their mass up to a critical value, as well as, decrease

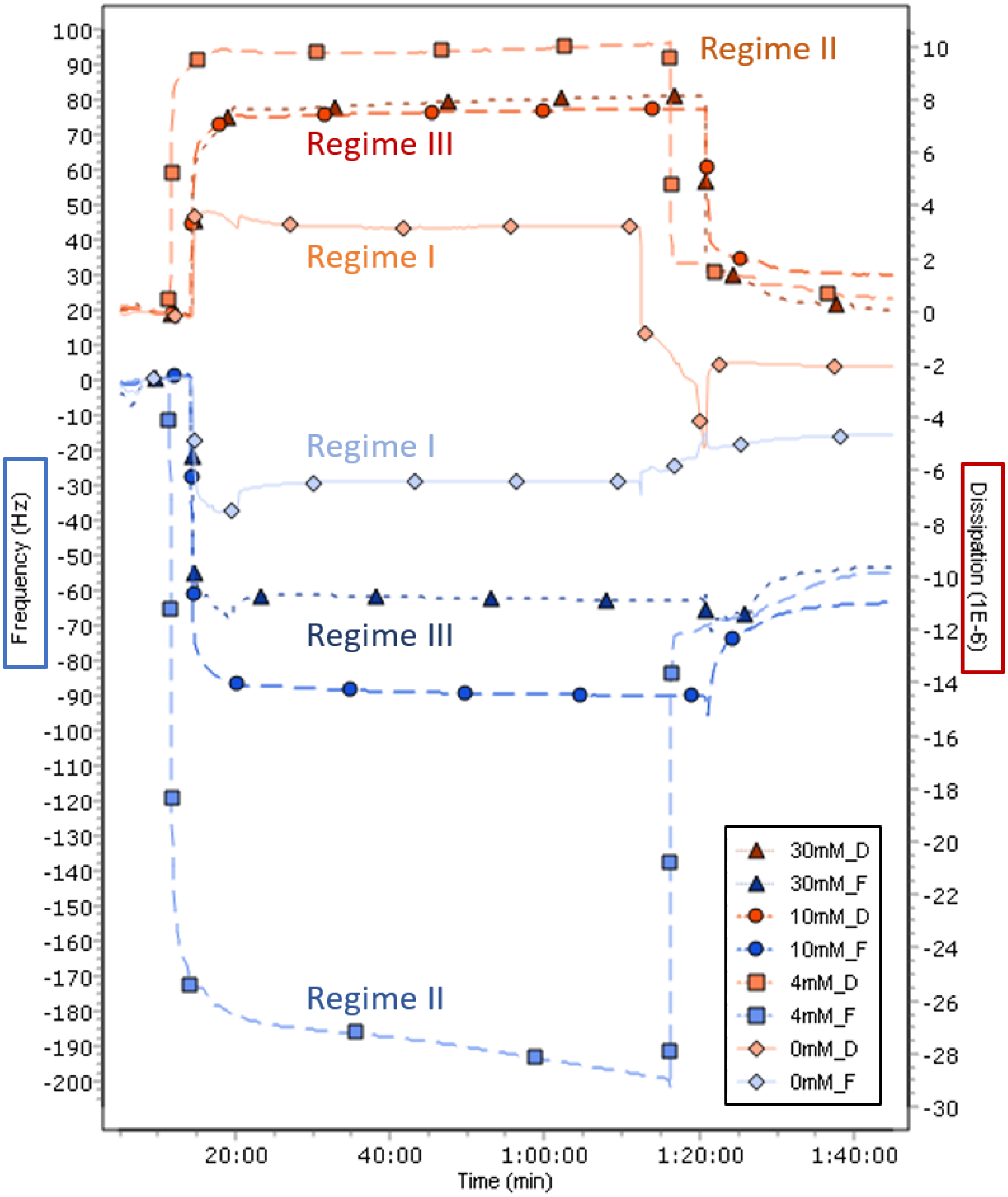

Figure 5: **Real-time protein adsorption.** 9th overtone of the raw QCM-D data showing the change in frequency (blue) and dissipation (red) upon (step 1) the additional of protein/salt solution and (step 2) rinsing with water at different salt concentrations at 20 mg/ml c_p and 40°C. Note: During solution exchanges the pump creates spikes during activation and shut-down in the data, which should be ignored.

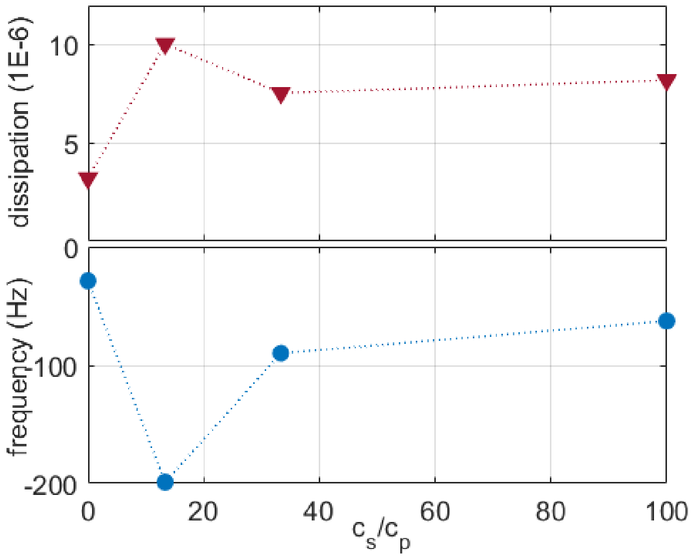

Figure 6: **Raw QCM-D data.** Absolute values of frequency (blue) and dissipation (red) of the 9th overtone of BSA/YCl_3 at 40°C.

in dissipation. A stiffer and and much thinner layer is left at the interface. This observation can also be visualised by plotting the absolute values of D and F in Fig. 6.

Through the fitting of the data with a viscoelastic model, the thickness and/or mass of the adsorbed proteins, its viscosity and elasticity can be extracted. In Fig. 4 (main text), d_{QCM-D} at 40°C and in Fig. 7 d_{QCM-D} at 20°C is illustrated. The constants used in the fitting process can be found in Tab. II. The trends mentioned above are also reflected in the extracted viscosity parameter in Fig. 7. While the viscosity parameter is not very reliable on an *absolute* scale, it is an interesting and useful quantity to compare on a *relative* scale. The more diffuse layer also seem to have a higher viscosity.

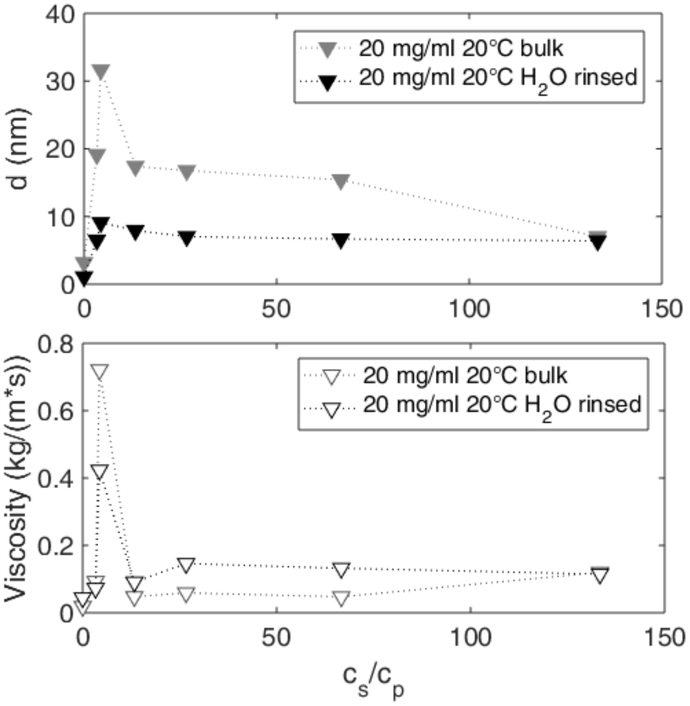

Figure 7: **Temperature-dependent adsorption behaviour.** QCM-D measurements of the adsorbed amount of proteins at the solid liquid interface at 20 mg/ml and 20°C. The top image illustrates the adsorption behaviour and its dependence on c_s. The black data points show d after flushing the cell with H_2O, thus, the irreversibly bound proteins, which show no re-entrant adsorption any more. Its correlated viscosity is plotted in the bottom image.

Table II: **Material properties.** Fitting constants for QCM-D data modelling [5–8].

**Material**	**Density [g/L]**	**Viscosity [kg/(m*s)]**
H_2O (25°C)	997	0.00089
H_2O (40°C)	992	0.00065
BSA (20 mg/ml)	1003.4	0.0011
BSA (50 mg/ml)	1013.2	0.0013
BSA (powder)	1320	-
Adsorbed monolayer (1 ML)	1192.5	-

LIST OF ABBREVIATIONS

B_2/B_2^{HS} - reduced second virial coefficient

BLG - beta-lactoglobulin

BSA - bovine serum albumin

c^* - first phase transition

c^{**} - second phase transition

c_p - protein concentration

c_s - salt concentration

d - thickness of adsorbed protein

d_{assoc} - thickness/amount of associated water within protein layer

d_{EM} - equivalent to d; thickness of adsorbed proteins determined with ellipsometry

d_{rinsed} - thickness of irreversibly bound proteins

d_{QCM-D} - thickness of adsorbed proteins with associated water

DFT - classical density functional theory

ε_B - binding energy between patch and ion

ε_{PP} - binding energy between protein/patch and protein/patch

ε_{UO} - binding energy between occupied patch and unoccupied patch

FMT - fundamental measure theory

$\mathcal{F}[\rho]$ - functional of the intrinsic free energy

LLPS - liquid-liquid phase separation

M - amount of patches on hard sphere

μ_S - salt chemical potential

$\Omega[\rho]$ - functional of the grand potential

QCM-D - quartz-crystal microbalance with dissipation

RC - re-entrant condensation

R_p - hard sphere radius

$\rho(z)$ - protein density profile

SAM - self-assembled monolayer

SiO_2 - silicon dioxide

T - temperature

Θ - occupation probability of a patch

$V_{\text{ext}}(\mathbf{r})$ - arbitrary external potential

$V_{\text{wp}}(z)$ - short-ranged wall-potential potential

YCl_3 - yttrium chloride

z - height of adsorbed layer

---

[1] Matsarskaia, O. *et al.* Cation-induced hydration effects cause lower critical solution temperature behavior in protein solutions. *J. Phys. Chem. B* **120**, 7731–7736 (2016).

[2] Zhang, F. *et al.* Reentrant condensation of proteins in solution induced by multivalent counterions. *Phys. Rev. Lett.* **101**, 148101 (2008).

[3] Roosen-Runge, F., Heck, B. S., Zhang, F., Kohlbacher, O. & Schreiber, F. Interplay of pH and Binding of Multivalent Metal Ions: Charge Inversion and Reentrant Condensation in Protein Solutions. *J. Phys. Chem. B* **117**, 5777–5787 (2013).

[4] Feiler, A. A., Sahlholm, A., Sandberg, T. & Caldwell, K. D. Adsorption and viscoelastic properties of fractionated mucin (BSM) and bovine serum albumin (BSA) studied with quartz crystal microbalance (QCM-D). *J. Colloid Interface Sci.* **315**, 475 – 481 (2007).

[5] Heinen, M. *et al.* Viscosity and diffusion: crowding and salt effects in protein solutions. *Soft Matter* **8**, 1404–1419 (2012).

[6] Rheosense, I. Application note: Viscosity measurement of a model protein solution of BSA. Tech. Rep., VROC-APP-04 (2008).

[7] Crittenden, J. C., Trussell, R. R., Hand, D. W., Howe, K. J. & Tchobanoglous, G. *MWH's water treatment: principles and design* (John Wiley & Sons, 2012).

[8] Korson, L., Drost-Hansen, W. & Millero, F. J. Viscosity of water at various temperatures. *J. Phys. Chem.* **73**, 34–39 (1969).
